# A revision of the genus *Ufeus* Grote with the description of a new species from Arizona (Lepidoptera, Noctuidae, Noctuinae, Xylenini, Ufeina)

**DOI:** 10.3897/zookeys.264.3526

**Published:** 2013-02-06

**Authors:** J. Donald Lafontaine, J. Bruce Walsh

**Affiliations:** 1 Canadian National Collection of Insects, Arachnids, and Nematodes, Biodiversity Program, Agriculture and Agri-Food Canada, KW Neatby Bldg., C.E.F., Ottawa, Ontario, Canada K1A 0C6; 2 Dept of Ecology and Evolutionary Biology, Biosciences West, University of Arizona Tucson, AZ USA 85721; Research Associate: McGuire Center for Lepidoptera and Biodiversity, Florida Museum of Natural History, University of Florida, Gainesville, Florida, USA

**Keywords:** Taxonomy, Xylenini, Ufeina, *Ufeus*, Arizona

## Abstract

The genus *Ufeus* Grote is revised to include five species including *Ufeus felsensteini*,**sp. n.** in southern Arizona. A key to species, descriptions, illustrations of adults and genitalia are included.

## Introduction

The genus *Ufeus* Grote is an isolated genus that was included in the subfamily Noctuinae (s.s.) for almost a century (e.g., Hampson 1903) because of the presence of spiniform setae on the middle and hind tibiae. Crumb (1956) proposed a separate subfamily for the genus based on two peculiarities of the larvae, two L-setae on abdominal segment 9, and prolegs with more than 50 crochets. The Ufeinae were downgraded to a tribe of the Noctuinae by Franclemont and Todd (1983), raised again to subfamily level by Kitching and Rawlins (1998), and more recently, included within the Xylenini by Mitchell et al. (2006). Following the results of Mitchell (op. cit.) the group was treated as Ufeina, a subtribe of the Xylenini, by Lafontaine and Schmidt (2010) within an expanded concept of Noctuinae. In addition to the molecular results, additional characters that support placement in the Xylenini are: the presence of spiniform setae on the middle and hind tibiae in some genera (e.g., *Rhizagrotis* Smith, *Sutyna* Todd, *Fishia* (Grote), genera also included in the Noctuinae by Hampson 1903); larvae feeding mainly on woody plants; adults overwintering; adults with lashes in front of eye at base of antenna. Recently, Wagner et al. (2011) treated *Ufeus* as a tribe separate from the Xylenini based on the peculiarities of the larvae found by Crumb (op. cit.). However, with *Ufeus* nested within the Xylenini, such a move also would require the other subtribes of the Xylenini to be raised to tribal status, and this would impact on the status of the other tribes of the subfamily Noctuinae. As a result, we retain the *Ufeina* as a subtribe of the Xylenini in order to preserve the phylogenetic associations of the genus.

## Materials and methods

### Repository abbreviations

Specimens were examined from the following collections:

**AMNH** American Museum of Natural History, New York, New York, USA.

**BMNH** The Natural History Museum (statutorily, British Museum (Natural History), London, UK.

**CNC** Canadian National Collection of Insects, Arachnids, and Nematodes, Ottawa, Ontario, Canada.

**CUIC** Cornell University Insect Collection, Ithaca, New York, USA.

**FMNH** The Field Museum, Chicago, Illinois, USA.

**JBW** Personal collection of J. Bruce Walsh, Tucson, Arizona, USA.

**NYSM **New York State Museum, Albany, New York, USA.

**USNM **National Museum of Natural History (formerly, United States National Museum), Washington, District of Columbia, USA.

**Dissecting methods and genital terminology**. Dissection of genitalia and terms for genital structures and wing markings follow Lafontaine (2004).

#### Ufeus

Grote, 1873

http://species-id.net/wiki/Ufeus

##### Type species.

*Ufeus satyricus* Grote, 1873, by original designation.

##### Diagnosis.

**Adults.** Males typically smaller and paler than females (forewing length 15–20 mm, males, 17–23 mm, females). Vestiture of palpi, head, and thorax of long hair-like scales, without evident tufting. *Head* **–** Male antenna constricted between segments with long setae tending to form a tuft on each side of each segment (*Ufeus satyricus*) or filiform or very slightly constricted with setae minute in *Ufeus faunus* Strecker, *Ufeus felsensteini*, sp. n., *Ufeus hulstii* Smith, and *Ufeus plicatus* Grote. Female antenna filiform, minutely setose ventrally. Eye slightly reduced, smooth, without surface hair. Labial palpus porrect, apical segment usually about ½ as long as second segment. *Thorax* **–** *Wings*: Forewing ground color typically gray brown to reddish brown; pattern reduced to small elongated remnants of reniform and orbicular spots, faint dentate postmedial line, and darker shading and wedge-shaped spots in terminal area; an elongated black streak through position of reniform and orbicular spots in most species, especially in females. Hindwing translucent white to dark fuscous, a darker discal spot in most species; a dark postmedial line in *Ufeus satyricus*. *Legs*:a few sclerotized spiniform setae on middle and hind tibiae proximal to apical spurs in most specimens; spurs relatively short with longer spur in each pair about as long as width of tibia. Basitarsus with three ventral rows of spiniform setae, increased to a fourth row near apex; central row of setae tending to duplicate into two or three irregular rows on tarsal segments 2–5. *Abdomen* **–** Base of abdomen without basal abdominal brushes; abdomen clothed with long hair-like setae overlaying flat broad setae underneath; abdomen dorso-ventrally flattened, especially in females. **Male genitalia –** Uncus typically expanded preapically with apex flattened, tapered, heavily sclerotized, and forked (apex rounded in *Ufeus satyricus*). Tegumen variable, from about as wide as vinculum in *Ufeus satyricus* but much broader than vinculum in *Ufeus felsensteini*. Valve with sacculus usually slightly more than ½ length of valve; valve slightly constricted beyond sacculus, broadly rounded at apex; without corona or digitus; clasper in *Ufeus satyricus* arising on ventral margin of valve at apex of sacculus, gradually widening in oblique angle across valve, then forming a flattened twisted arm above dorsal margin of valve curving posteriorly almost to valve apex; clasper in other four species in middle of valve beyond sacculus with base forked extended to ventral margin of valve and dorsal margin of sacculus; distal to base clasper slightly tapered, but expanded and spatulate apically. Aedeagus about 10 × as long as wide in *Ufeus satyricus* and vesica a slender curving tube about ½ as long as aedeagus; in other species aedeagus 4–6 × as long as wide and vesica about as long as aedeagus and with 1–3 fields of spine-like cornuti on short diverticula. **Female genitalia –** Corpus bursae thin and membranous, rounded or oval, without obvious signa, except in *Ufeus plicatus* and *Ufeus hulstii*; posterior part of corpus bursae tapered directly into ductus bursae (*Ufeus satyricus*), or covered with striated sclerotized bands (other species), giving rise to appendix bursae in three species. Ductus bursae heavily sclerotized, even in width throughout (*Ufeus satyricus*), or expanded into broad posterior pouch (other four species). Anterior apohyses about as long as abdominal segment eight and ½ × as long as posterior apophyses (*Ufeus satyricus*), or ovipositor telescopic with anterior apohyses about 3 × as long as abdominal segment eight and ½ × as long as posterior apophyses. Anal papillae rounded, lightly sclerotized, covered with long hair-like setae.

##### Larva and habits.

The larva is characterized by the large number of crochets (> 50) on each proleg, and the presence of two L setae on abdominal segment 9 (Crumb 1956, Wagner et al. 2011). The larvae are said to hide by day under strips of bark in *Ufeus plicatus* (i.e., Wagner et al. 2011) and *Ufeus faunus* (i.e., Crumb 1956), and adults of *Ufeus satyricus* are reported to do this also (i.e., Forbes 1954). It is likely that all species share this habit and also would explain the tendency for adults to be dorsoventrally flattened. The larvae, where known, feed on poplar and willow and may prefer large trees where there is abundant loose bark near the base of the tree. The large number of crochets in the larvae may be an adaptation to feeding on poplar leaves. The petiole on a poplar leaf is laterally flattened, making it hard to hold on to and causes it to shake – even in a light breeze.

**Figures 1–14.**
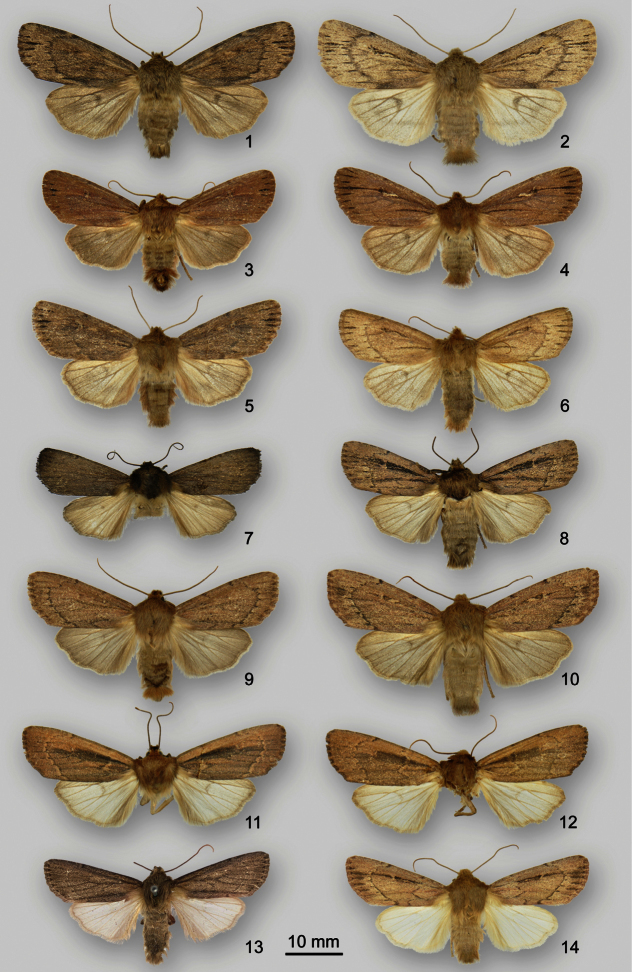
*Ufeus* adults **1** *Ufeus satyricus satyricus* ♂, New Brunswick, Fredericton **2** *Ufeus satyricus satyricus* ♀, New Brunswick, Fredericton **3** *Ufeus satyricus sagittarius* ♂, California, San Diego Co., Laguna Mts **4** *Ufeus satyricus sagittarius* ♀, California, Plumas Co., Johnsville **5** *Ufeus satyricus sagittarius* ♂, Arizona, Santa Cruz Co., Patagonia Mts **6** *Ufeus satyricus sagittarius* ♀, Montana, Bozeman **7** *Ufeus plicatus* ♂, Nebraska, Omaha **8** *Ufeus plicatus* ♀, Quebec, Laval **9** *Ufeus hulstii* ♂, British Columbia, Watch Peak, 50°29'N, 116°18'W **10** *Ufeus hulstii* ♀, British Columbia, Gott Peak, 50°21'N, 122°08'W **11** *Ufeus felsensteini* holotype ♂, Arizona, Pima Co., Santa Catalina Mts **12** *Ufeus felsensteini* paratype ♀, Arizona, Pima Co., Santa Catalina Mts **13** *Ufeus faunus* holotype ♂, New Mexico **14** *Ufeus faunus* ♀, California, Mojave Desert near Llano.

##### Key to North American species of *Ufeus*

**Table:** 

1	Forewing with black intervenal dashes in subterminal area; hindwing with darker postmedial line, especially obvious on underside; male genitalia with clasper above dorsal margin of valve, tapered toward apex; ovipositor short (posterior apophyses 2 × as long as abdominal segment eight)	*Ufeus satyricus*
–	Forewing with subterminal area clear, or with diffuse dark shading; hindwing unicolorous, without darker postmedial line (except occasionally in *Ufeus plicatus*); male genitalia with clasper on inner surface of valve, apically spatulate; ovipositor telescoping (posterior apophyses 4–7 × as long as abdominal segment eight)	2
2	Hindwing fuscous; uncus in male genitalia with large preapical bulge dorsally giving profile like a duck’s head; corpus bursae with two signa	3
–	Hindwing white and translucent, sometimes with a slight smoky or reddish sheen; uncus with at most a slight preapical bulge; corpus bursae without signa	4
3	Clasper in male genitalia about ½ × as wide as valve; patch of spike-like cornuti on ventral side of vesica much stouter than those on dorsal side; occurring from southern Quebec to Pennsylvania westward to eastern Nebraska	*Ufeus satyricus plicatus*
–	Clasper in male genitalia wider, ⅔– ¾ × as wide as valve; patch of spike-like cornuti on ventral side of vesica similar to those on dorsal side; occurring from foothills of Alberta and Colorado westward	*Ufeus hulstii*
4	Forewing reddish brown with diffuse dark streak extending from wing base to postmedial line; vesica in male genitalia elongated with three patches of spike-like cornuti; posterior rugose part of corpus bursae as wide as anterior part and lobed	*Ufeus felsensteini*
–	Forewing buffy brown, sometimes with a narrow black line through reniform and orbicular spots; vesica in male genitalia rounded with two patches of spike-like cornuti; posterior rugose part of corpus bursae long and narrow, about ¼ × as wide as anterior part	*Ufeus faunus*

## Systematics

### Ufeus satyricus

Grote, 1873

http://species-id.net/wiki/Ufeus_satyricus

Figs 1–6 15 20

Ufeus satyricus Grote, 1873: 101.
Asterocampus barometricus Goossens, 1881: 380.
Ufeus sagittarius Grote, 1883: 31.
Ufeus electra Smith, 1908: 99.
Ufeus unicolor ab. coloradica Strand, [1916]: 146. Unavailable infrasubspecific name.
Ufeus unicolor ssp. coloradica McDunnough, 1938: 68. Validation of *coloradica*.

#### Type material.

*Ufeus satyricus*: **syntypes** 2 ♀. [London], Ontario, Canada [lost]; Albany New York, NYSM. *Asterocampus barometricus*: Canada [type lost]; original description diagnostic for synonym of *Ufeus satyricus*. *Ufeus sagittarius*: **holotype** ♀. California, USNM. *Ufeus electra*: lectotype ♀, Oregon, AMNH, designated by Todd (1982). *Ufeus unicolor* ssp.* coloradica*: **syntype** ♂. Colorado, BMNH.

#### Other material examined and distribution.

**Canada:** Alberta, British Columbia, Manitoba, New Brunswick, Nova Scotia, Ontario, Quebec, Saskatchewan. **USA:** Arizona, California, Colorado, Montana, New York, Oregon, Utah, Washington.

#### Diagnosis.

*Ufeus satyricus* is abundantly distinct structurally from all other species in the genus. Superficially, adults in eastern North America can be distinguished from the largely sympatric *Ufeus plicatus* by the pale-brown forewings, extensively dusted with black scales, and the prominent black shading on the veins, discal spot, and postmedial line on the hindwing. Males are smaller and darker than females (forewing length 15–22 mm in males, 19–24 mm in females). Adults from western North America differ from those from the East in having darker reddish-brown forewings with the postmedial line less prominent, and the hind wing in the male is dark fuscous, obscuring the postmedial line on the upper surface of the wing. Western specimens are easily confused with those of *Ufeus hulstii*, which have similar reddish-brown forewings. The black streaks in the subterminal area and the less prominent postmedial line on the forewing, and the postmedial line on the hindwing, at least on the underside of the wing, allow specimens of *Ufeus satyricus* to be distinguished from those of *Ufeus hulstii* without dissection. Western populations of *Ufeus satyricus* are segregated as *Ufeus satyricus* ssp. *sagittarius*. Intermediate populations are in Wyoming and Colorado. The **male genitalia** of *Ufeus satyricus* are characterized by the dorsal clasper and the long slender aedeagus and vesica. In the **female genitalia** the corpus bursae is rounded and extends directly into the long sclerotized ductus bursae. The anterior and posterior apophyses are relatively short (as described in the generic diagnosis), so the ovipositor is not telescoping.

#### Distribution and biology.

*Ufeus satyricus* occurs across central and southern Canada from the Atlantic to the Pacific where large poplar trees occur and as far south in the east as Pennsylvania and Illinois. In the west it occurs as far south as southern Arizona and California. Adults emerge from the pupae in the summer and overwinter as adults, but they are mostly collected between late August and early May, even during mild spells in mid-winter. Most records are in October and November in the fall and March and April in the spring. Crumb 1956 reports finding and rearing larvae on cottonwood in western United States. The species is arranged in two subspecies.

### Ufeus satyricus satyricus

Grote, 1873

http://species-id.net/wiki/Ufeus_satyricus_satyricus

Figs 1, 2 15

Ufeus satyricus Grote, 1873: 101.
Asterocampus barometricus Goossens, 1881: 380.

#### Remarks.

In *Ufeus satyricussatyricus* the forewing in both sexes is pale brown, heavily speckled with black, and the postmedial line is prominent and dentate. The hindwing in the male is a mottled fuscous with darker fuscous shading on the veins, discal spot, and a diffuse postmedial line. The hindwing in the female is similar to that of the male except the ground color of the wing is pale buffy brown. The nominate subspecies occurs from eastern North America westward to the foothills of the Rocky Mountains.

### Ufeus satyricus sagittarius

Grote, 1883

http://species-id.net/wiki/Ufeus_satyricus_sagittarius

Figs 3–6 20

Ufeus sagittarius Grote, 1883: 31.
Ufeus electra Smith, 1908: 99.
Ufeus unicolor ab. coloradica Strand, [1916]: 146. Unavailable infrasubspecific name.
Ufeus unicolor ssp. coloradica McDunnough, 1938: 68. Validation of *coloradica*.

#### Remarks.

In *Ufeus satyricussagittarius* sexual dimorphism is much more obvious than in the eastern subspecies. In the male the forewing is a shiny dark reddish brown with the maculation obscure except for a trace of pale streaks representing the reniform and orbicular spots and a hint of a darker postmedial line. The hindwing is dark fuscous, usually obscuring the postmedial line and discal spot. In the female the forewing is a paler reddish brown with a black streak between, above, or through the pale streaks representing the reniform and orbicular spots, the postmedial line is prominent, and usually there are numerous black streaks in the subterminal area. The hindwing is pale fuscous with the discal spot and postmedial line contrastingly darker. Unlike subspecies satyricus, the two sexes of *sagittarius* differ markedly in size with forewing length averaging 17. 6 mm (*n*=20) in males and 19.7 mm (*n*=20) in females. The two sexes of this subspecies frequently are sorted as two separate species in collections. Subspecies *sagittarius* occurs from the eastern edge of the Rocky Mountains in Alberta, Montana, and Colorado westward to the Pacific Coast.

**Figures 15–19.**
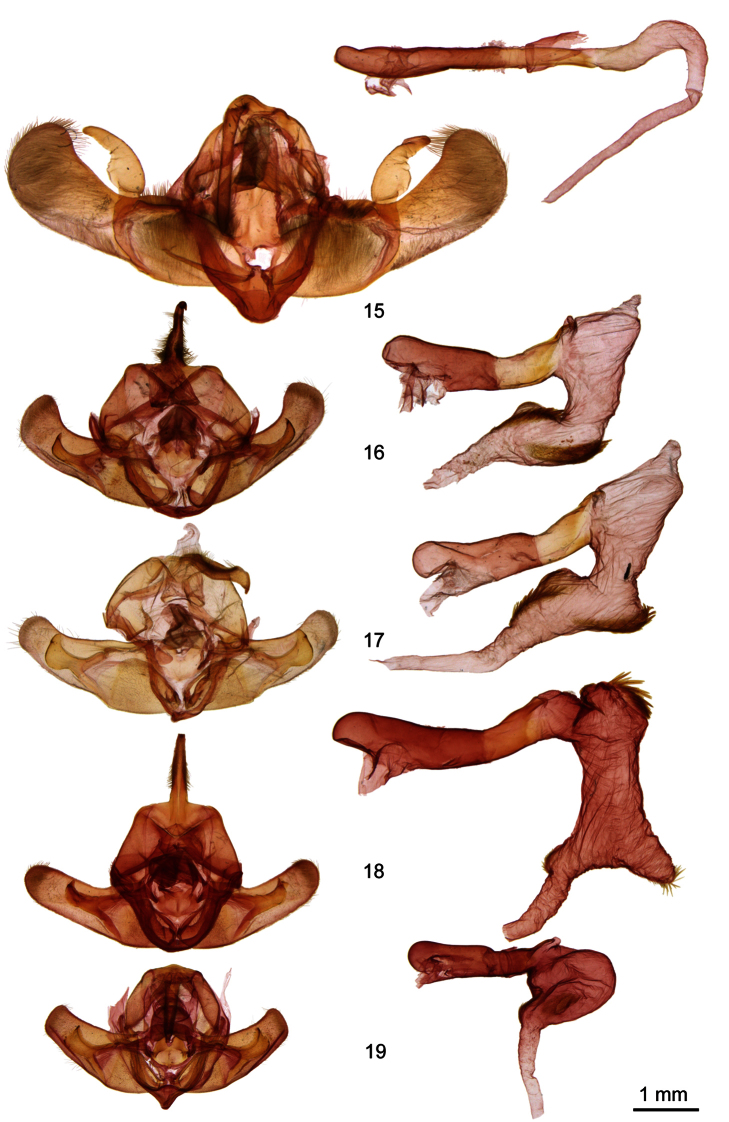
*Ufeus* male genitalia. **15** *Ufeus satyricus* **16** *Ufeus plicatus* **17** *Ufeus hulstii* **18** *Ufeus felsensteini* **19** *Ufeus faunus*.

### Ufeus plicatus

Grote, 1878

http://species-id.net/wiki/Ufeus_plicatus

Figs 7, 8 16 21

Ufeus plicatus Grote, 1873: 102.
Ufeus unicolor Grote, 1878: 179.

#### Type material.

*Ufeus plicatus*: **holotype** ♂. Illinois [type lost but description diagnostic]. *Ufeus unicolor*: **holotype** ♂. Illinois, BMNH.

#### Other material examined and distribution.

**Canada:** Ontario, Quebec. **USA**: Illinois, Iowa, Nebraska.

#### Diagnosis.

*Ufeus plicatus* occurs sympatrically with *Ufeus satyricus* in northeastern North America but can be distinguished from it by the darker, more even, somewhat glossy, dark reddish-brown or blackish-brown color of the forewing in males and the reddish-brown color of the forewing with a long blackish streak extending from the wing base through the orbicular and reniform spots into the subterminal area in females. In both sexes the hindwing is evenly colored light fuscous with at most a slight trace of a discal spot and postmedial line. Males average only slightly smaller than females (forewing length 16–19 mm in males, 17–20 mm in females). *Ufeus plicatus* is most closely related to *Ufeus hulstii*, which occurs from the Rocky Mountains westward. In addition to range, adults can superficially be distinguished from those of *Ufeus hulstii* by the darker color of the forewing in males, and the more extensive dark streak through the forewing cell in females. In the **male genitalia** of *Ufeus plicatus* the clasper is positioned on the inner surface of the valve with the expanded apical part about ½ × as wide as the valve (⅔– ¾ × as wide in *Ufeus hulstii*); the vesica has two elongated patches of spike-like setae; the setae in ventral patch (near the aedeagus) are much stouter than those in the dorsal patch (in *Ufeus hulstii* the setae are similar in size in both patches). In the **female genitalia** the corpus bursae is ⅓–½ × as wide as its length, and has a large, rugose sclerotized appendix bursae posteriorly. The sclerotized part of the ductus bursae is wedge shaped, wide posteriorly and evenly tapered anteriorly. The ovipositor is telescoping with the anterior apophyses about 4 × as long as abdominal segment eight and the posterior apophyses about 7 × as long. The corpus bursae is narrower, about 1/3 × as wide as its length, and has a smaller rugose sclerotized appendix bursae posteriorly. The sclerotized part of the ductus bursae is narrow posteriorly, widens anteriorly to ¼ × wider, before tapering anteriorly. The ovipositor is telescoping, as *Ufeus plicatus*.

#### Distribution and biology.

*Ufeus plicatus* is an extremely rarely-collected species. Until recently the few specimens known were only from the mid-west, mostly from Illinois with a few records from Iowa, Minnesota, Missouri, and Nebraska. This led Forbes (1954) to suggest that the type locality of Philadelphia, Pennsylvania, was almost certainly in error for Illinois. Recent collections of the species from southern Quebec (Handfield 2011) and Connecticut (Wagner et al. 2011) suggest that not only is Philadelphia a possibility, but that the species might be widespread in the Northeast as is its highly localized and specialized habitat. The species is associated with large poplars, especially eastern cottonwood (*Populus* *deltoides* Bartram ex Marsh.) growing in moist areas along rivers where there is abundant loose rotting strips of bark near the base of the tree. Larvae hide under the strips of bark during the day and the adults likely hide there also during the day and in the winter. According to Wagner et al. (2011) the eggs are laid in the spring with adults emerging in late spring and early summer, but mainly aestivating until the fall before becoming active. Adults have been recorded in all months except June, but most records are from October and November in the fall and March and April in the spring. The scarcity of adults, even in suitable habitats where they are known to occur, suggests they may not be strongly attracted to light.

### Ufeus hulstii

Smith, 1908

http://species-id.net/wiki/Ufeus_hulstii

Figs 9, 10 17 22

Ufeus hulstii Smith, 1908: 99.
Ufeus lura Dyar, 1914: 370, **syn. n.**

#### Type material.

*Ufeus hulstii*: **lectotype** ♂. Stockton, Utah, AMNH, designated by Todd (1982). *Ufeus lura*: **holotype** ♂. Mexico City, Mexico, USNM.

#### Other material examined and distribution.

**Canada:** Alberta, British Columbia. **Mexico:** Distrito Federal, Durango. **USA**: Alaska, Arizona, California, Colorado, Idaho, Montana, New Mexico, Nevada, Oregon, Utah, Washington.

#### Remarks.

*Ufeus hulstii* is the western counterpart of *Ufeus plicatus* and was treated as a subspecies of it for many years. Differences in external appearance, male and female genitalia, barcodes, and biology led to its recognition as a separate species by Lafontaine and Schmidt (2010), but they used the name *Ufeus electra* for it, a name that had been treated as a synonym of *Ufeus plicatus* by Franclemont and Todd (1983) and Poole (1989). Re-examination of the type material resulted in the name *Ufeus electra* being transferred to the synonymy of *Ufeus satyricus* and *Ufeus hulstii* being used for this species (Lafontaine and Schmidt 2011).

#### Diagnosis.

In *Ufeus hulstii* both sexes have an orange-brown forewing and fuscous hindwing with males averaging slightly darker than females. Most females of *Ufeus hulstii* have a dark streak through the orbicular and reniform spots, but the streak does not normally extend to the postmedial line or into the basal area of the wing. Although occasionally specimens of *Ufeus hulstii* are as small as those of *Ufeus plicatus* (16 mm), they are, on average, much larger with forewing lengths up to 22 mm in males and 23 mm in females. The **male genitalia** of *Ufeus hulstii* differ from those of *Ufeus plicatus* by the characters given in the key and in the diagnosis for *Ufeus plicatus*. The **female genitalia** of *Ufeus hulstii* are similar to those of *Ufeus plicatus*.

#### Distribution and biology.

*Ufeus hulstii* is widely distributed in western North America from central Alaska southward to south-central Mexico and from the Rocky Mountain foothills to the West Coast. The larvae are reported to feed on poplar, aspen, and willow with adults emerging in early summer (Crumb 1956). Like other species, the adults overwinter, but they also are more frequently collected during the summer months than other species.

**Figures 20–24.**
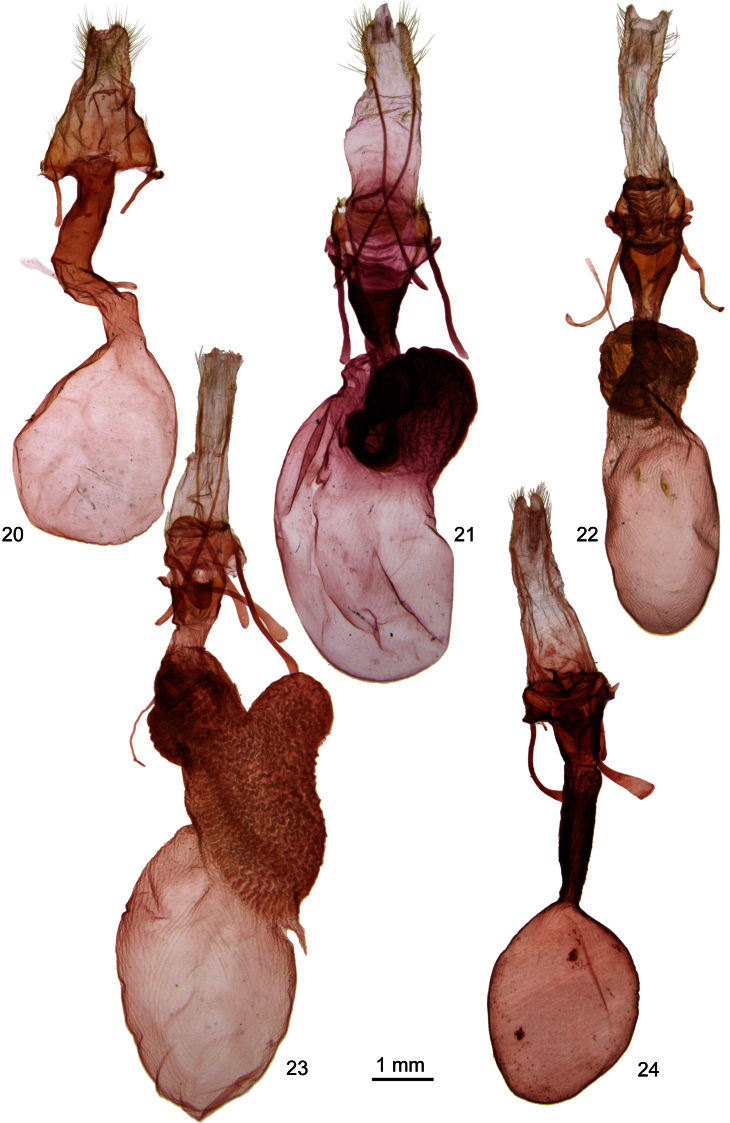
*Ufeus* female genitalia. **20** *Ufeus satyricus* **21** *Ufeus plicatus* **22** *Ufeus hulstii* **23** *Ufeus felsensteini* **24** *Ufeus faunus*.

### Ufeus felsensteini

Lafontaine & Walsh n. sp.

urn:lsid:zoobank.org:act:1BA7C2AE-95F3-4ECE-8579-FBCC525D6A32

http://species-id.net/wiki/Ufeus_felsensteini

Figs 11, 12 18 23

#### Type material.

**Holotype** ♂. Arizona, Pima Co., Santa Catalina Mts, Bear Wallow Road, 8000’, uv light trap, 18 May 2003, B. Walsh. CNC. **Paratypes:** 1 ♂, 3 ♀. Arizona, Pima Co., Santa Catalina Mts, Bear Wallow Road, 7800’, uv lights, pine forest, 21 May 2005, B. Walsh (1 ♂); USA, Arizona, Pima Co., Santa Catalina Mts, mile 5.5 Mt. Lemmon Hwy, 4400’, uv light trap, riparian/blue oak woodland, 16 Jan. 2005, B. Walsh (1 ♀); USA, Arizona, Pima Co., Santa Catalina Mts, Molino Canyon, 4,100’, mile 4.5 Mt Lemmon Hwy, uv light trap, riparian habitat, 1 Jan. 2012, B. Walsh (1 ♀); USA, Arizona, O. Bryant (1 ♀). Paratypes deposited in CNC, JBW.

#### Etymology.

The species name is in honor of Professor Joseph Felsenstein, who pioneered modern statistical methods in the reconstruction of phylogenies.

#### Diagnosis.

*Ufeus felsensteini*can be recognized by the reddish-brown forewing with the maculation obscure except for a prominent black dash from the wing base to the reniform spot, then continuing below the reniform spot to, or slightly past, the postmedial line, and by the translucent hindwing with a pearly-pinkish sheen. It is most closely related to *Ufeus hulstii*, both species having similar male and female genitalia, but in *Ufeus felsensteini* there is a cluster of long spike-like setae on the subbasal diverticulum of the vesica, not just on the two subapical diverticula as in *Ufeus hulstii*, and the uncus lacks the preapical dorsal lobe found in *Ufeus hulstii*. The female genitalia of *Ufeus felsensteini* have much more extensive rugose sclerotized banding than in other species, extending over the posterior part of the corpus bursae, appendix bursae, and anterior part of the ductus bursae.

#### Description.

**Adults.** Male and female similar in size, color, and maculation. Forewing length: 19–21 mm. **Head –** Male and female antennae with individual segments very slightly constricted between segments; minutely setose ventrally. Palpi and head mainly covered with reddish-brown scales, but with blackish-brown scales on frons and scattered blackish-brown scales on palpi. **Thorax** **–** Covered with reddish-brown scales; without tufting. *Legs*: Covered with pale reddish-brown scales with scattered dark-gray scales, especially on outer side of tibiae. Distal half of middle and hind tibia with 5–8 spiniform setae. Tarsi with three ventral rows of spiniform setae on basal half of basitarsus, increasing to four rows on apical half; 2^nd^–4^th^ tarsi with four ventral rows of spiniform setae, five rows on 5^th^ segment. *Wings*: Dorsal forewing reddish brown with maculation obscure except for slightly paler antemedial and postmedial lines, the former lined distally with black, and the latter slightly dentate and lined proximally with black; wing with an increasingly wide black streak extending from wing base to reniform spot, then continuing below reniform spot to, or slightly beyond, postmedial line; reniform and orbicular spots indicated by minute paler spots within dark forewing dash; terminal line concolorous with forewing, or with slight black wedge-shaped spots between veins. Fringe slightly checkered, with dark intervenal spots continuing on to fringe. Hindwing translucent white with a slight pearly-pink sheen; slightly darker fuscous shading on discal spot, wing margin, and fringe. **Male genitalia –** Uncus dorso-ventrally flattened, gradually tapering from base to apex with heavily-sclerotized, downward projecting plate at apex with pointed tip. Valve abruptly tapered from base, then apical half parallel-sided with rounded apex; corona and digitus absent; sacculus extending almost to middle of valve; clasper in middle of valve beyond sacculus with base forked, extending to ventral margin of valve and dorsal margin of sacculus; distal to base of clasper slightly tapered, but expanded and spatulate apically. Aedeagus about 6 × as long as wide with ventral extension at apex; vesica cylindrical with three diverticula each with a cluster of long spine-like cornuti, one subbasally with longest, stoutest cornuti, one preapically on outside with shorter, thinner cornuti, and one on inner side at apex with shortest, thinnest cornuti. **Female genitalia –** Corpus bursae bilobed, shaped like Figure 8, anterior lobe membranous, rounded; posterior lobe with diverticulum to right, and posterior extension leading to ductus bursae rugose, covered with twisted sclerotized bands. Ductus bursae about 0.15 × as long as corpus bursae with slightly tapered sclerotized plate in posterior half of ductus.

#### Distribution and biology.

*Ufeus felsensteini* is known only from the Santa Catalina Mountains of southeastern Arizona. The life history probably is similar to those of the other species of *Ufeus* with larvae associated with large cottonwoods; adults emerge in the spring and overwinter, mainly flying during the winter months.

### Ufeus faunus

Strecker, 1898

http://species-id.net/wiki/Ufeus_faunus

Figs 13, 14 19 24

Ufeus faunus Strecker, 1898: 9.

#### Type material.

**Holotype** ♂. New Mexico, USA, FMNH.

#### Other material examined and distribution.

**USA**: Arizona, California.

#### Diagnosis.

*Ufeus faunus* is the smallest and palest species in the genus. Forewing length is 15–17 mm in males and 17–19 mm in females. Both sexes have pale buffy-brown forewings with black defining a zigzagged antemedial line and a toothed postmedial line with dark shading and streaks in the outer half of the terminal area. In females usually there is a thin dark streak extending from the reniform spot to the postmedial line and, in extreme forms, from the antemedial line into the subterminal area. In both sexes the hindwing is translucent white with some buffy-brown shading on the terminal line. The **male genitalia** of *Ufeus faunus* differ from those of *Ufeus plicatus* and *Ufeus hulstii* in that the apex of the valve is truncated, not rounded, the apex of the clasper is notched, not rounded, and the vesica is globular, not elongated, with a dense patch of short sclerotized preapical cornuti on the right and a patch of longer cornuti at the apex. In the **female genitalia** the corpus bursae is gourd-shaped with a rounded membranous anterior part, and a long, narrow, almost neck-like posterior part with the surface rugose and sclerotized and the ductus seminalis arising dorsally at the posterior end. The ductus bursae is short, only 0.15 × as long as the two parts of the corpus bursae and almost entirely sclerotized. As in other members of the *Ufeus plicatus* group, the ovipositor telescopes.

#### Distribution and biology.

*Ufeus faunus* is known only from southwestern United States in a band extending from southwestern California to southern New Mexico. Crumb 1956 reports finding larvae under bark strips of cottonwood and willow near Superior, Arizona, in late March, with adults emerging in early May.

## Supplementary Material

XML Treatment for Ufeus

XML Treatment for Ufeus satyricus

XML Treatment for Ufeus satyricus satyricus

XML Treatment for Ufeus satyricus sagittarius

XML Treatment for Ufeus plicatus

XML Treatment for Ufeus hulstii

XML Treatment for Ufeus felsensteini

XML Treatment for Ufeus faunus
